# Bridging the quality chasm in maternal, newborn, and child healthcare in low- and middle-income countries

**DOI:** 10.1371/journal.pmed.1002465

**Published:** 2017-12-12

**Authors:** Lars Åke Persson

**Affiliations:** Department of Disease Control, London School of Hygiene & Tropical Medicine, London, United Kingdom

## Abstract

In a Perspective, Lars Åke Persson discusses the need to focus on quality of care to improve maternal, newborn, and child healthcare.

At the end of the Millennium Development Goal era, extreme poverty had been halved, and mortality in children below 5 years of age had been reduced from 90 per 1,000 live births in 1990 to 43 per 1,000 in 2015 [1]. This decrease in child deaths was the result of concerted global efforts to increase the coverage of evidence-based interventions [2]. Emphasis was primarily given to post-neonatal, child, and maternal deaths. Neonatal survival was added to the agenda after a few years [3], as was equity in child survival between and within countries [4]. The Sustainable Development Goals include targets for maternal, newborn, and child health under the umbrella of universal health coverage by 2030, where the quality of care and health equity are fundamental components [5]. These targets are aligned with global action plans to end preventable maternal mortality and save neonatal lives. In some countries, the rapid shift towards facility-based deliveries has increased neonatal mortality [6,7]. These unexpected results are most likely due to a poor quality of services provided. To improve maternal and neonatal survival, quality of care must be strengthened when facility-based deliveries increase [8].

## What is quality of care?

WHO describes quality of care, as “the extent to which healthcare services provided to individuals and patient populations improved desired outcomes. In order to achieve this, healthcare must be safe, effective, timely, efficient, equitable, and people-centred” [9]. Three decades ago Avedis Donabedian developed a conceptual framework in which quality of care was classified under 3 categories: structure (material and human resources and organizational structure), process (giving and receiving care), and outcome (effects on health status) [10].

This week in *PLOS Medicine*, Hannah Leslie and colleagues show that the facility infrastructure was poorly associated with the process, that is, the clinical quality of care provided to patients [11]. This cross-sectional assessment was based on 4,300 facilities in Haiti, Kenya, Malawi, Namibia, Rwanda, Senegal, Tanzania, and Uganda between 2007 and 2015. A well-equipped facility could provide inadequate care, and a facility scoring low on readiness could deliver care of good quality. This finding does not imply that service readiness is unimportant; equipment is always required to measure an expectant mother’s blood pressure. The analyses did not manage to demonstrate a minimum threshold of facility infrastructure needed for the provision of good care. The cross-sectional design of the study may be a limitation, as it does not capture the dynamics of the structural readiness over time. There may also be issues related to range and validity of available data on input and process. Still, the paper shows that the health facility infrastructure tells us very little about the quality of the maternal, newborn, and child health services provided in these settings.

## How should quality of care be assessed and promoted?

The framework suggested by WHO for improving the quality of care for mothers and newborns includes 8 components aligned to the health-system building blocks that should be assessed, improved, and monitored [9]. So far the existing tools and information systems are not adequate for measuring quality of care [12]. Measures do not reflect the process of care, and the experiences of patients are rarely represented. In a recent analysis of 68 quality checklists from a wide range of low- and middle-income countries, the indicators mainly focused on facility infrastructure and availability of resources [13]. In a metareview of almost 100 systematic reviews of interventions to improve the quality of care, the facilitators and barriers identified were in the domains of information, patient-population engagement, leadership, regulations and standards, organizational capacity, models of care, communication, and satisfaction [14]. There are few tools available to measure these facilitators and barriers; the Context Assessment for Community Health (COACH) tool is one of very few questionnaire instruments that has been developed and validated in low- and middle-income countries and includes context indicators in similar domains as the metareview [15].

During the past 3 decades the global health community has focused on coverage of evidence-based maternal, newborn, and child health services. With the new global goals and commitment up to 2030, it is essential that the quality of care dimension be added to the agenda. The paper by Leslie and colleagues reminds us that accurate measurements are needed that reflect the context, the processes of giving and receiving care, and the effects on the health status of patients and populations. This is needed to reach the new maternal, newborn, and child health goals.
